# Gestational gigantomastia—a rare entity complicated by life-threatening haemorrhage

**DOI:** 10.1093/jscr/rjab050

**Published:** 2021-04-14

**Authors:** Carlos Neblett, Rajeev Venugopal, Miguel Johnson, Derek Mitchell, Vojtech Kunc

**Affiliations:** 1 Division of Plastic Surgery, Department of Surgery, Radiology, Anaesthesia and Intensive Care & Emergency Medicine, University Hospital of the West Indies, Mona, Jamaica; 2 Division of General Surgery, Department of Surgery, Radiology, Anaesthesia and Intensive Care & Emergency Medicine, University Hospital of the West Indies, Mona, Jamaica; 3 Clinic of Trauma Surgery, Masaryk Hospital, Usti nad Labem, Czech Republic

## Abstract

Gestational gigantomastia is a psychologically and physically debilitating disease of unknown aetiology. Underlying diseases that present as gigantomastia should be excluded by a thorough workup. Most cases respond to the preferred approach: conservative management, as foetal viability and well-being is of significant importance. However, in those cases where the maternal mortality is at risk, the surgical approach is preferred. Life-threatening haemorrhage may occur and early recognition and treatment is paramount to outcome. A case of gestational gigantomastia complicated by life-threatening haemorrhage is presented and discussed.

## INTRODUCTION

Gestational gigantomastia (GG) is a rare idiopathic clinical condition, which manifests itself by characteristically exacerbated incapacitating breast hypertrophy during pregnancy [1]. Gigantomastia is defined as breast weight over 1.5 kg per or 3% of the patient’s total body weight [2]. GG was first reported by Palmuth in 1648 with fewer than 100 cases reported in the literature [1]. Reported incidence range from 1 in 28 000 to 1 in 100 000 pregnancies [3]. The authors herein discuss a case of this rare entity with an unusual complication that was managed at the University Hospital, a regional tertiary centre for Plastic and Reconstructive Surgery in the Caribbean. To the best of our knowledge, this case represents the first of such published report in the region.

**Figure 1:**
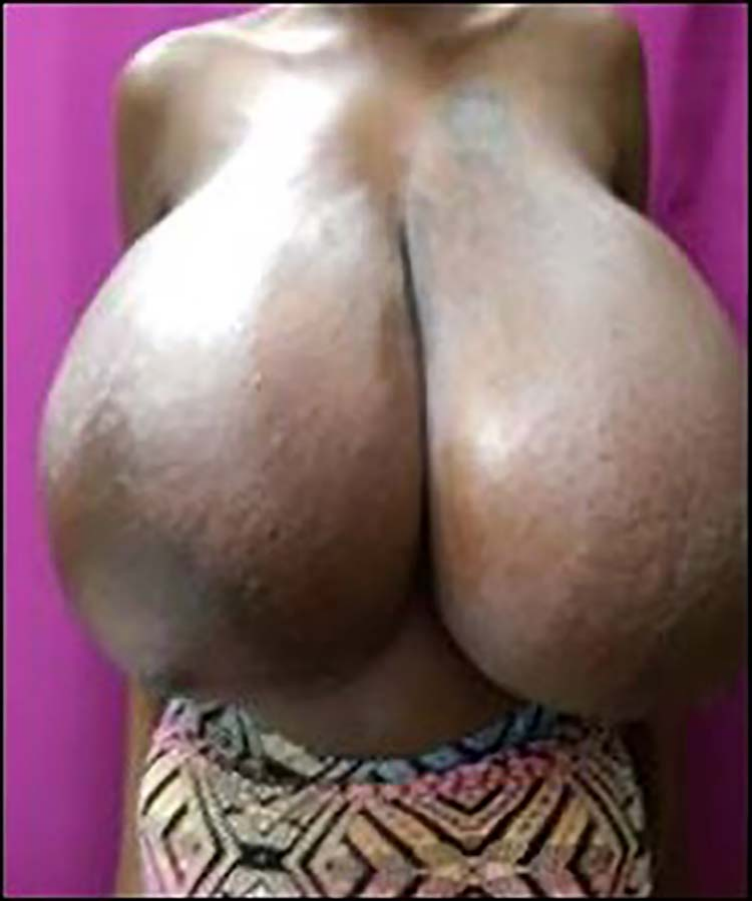
Photograph showing bilateral gigantomastia L > R in 22/40 gestational age patient.

## CASE

A 34-year-old woman with no chronic illnesses presented to the plastic surgery service at a gestational age (GA) of 22/40. She was gravida 4 para 2 + 1 with two prior live spontaneous vaginal deliveries and a miscarriage with neither any prior personal nor family history of breast pathology.

In her first trimester at 12/40 gestation, there was sudden and rapid progressive bilateral breast enlargement affecting the right side to a greater degree than the left. The increasing breast size was associated with chest and back pain, weight loss and distension of the veins of the breasts (Fig. 1). There was no associated nipple discharge.

Ultrasound evaluation demonstrated solid and cystic lesions bilaterally with associated left axillary adenopathy. Core needle biopsy of the left breast revealed psuedoangiomatous stromal hyperplasia. She had slightly elevated hormone levels of oestradiol 2557 pg/ml (*N* 188–2497 pg/ml) and prolactin 215 ng/ml (*N* 36–213 ng/ml) and was commenced on oral bromocriptine 2.5 mg twice daily.

**Figure 2:**
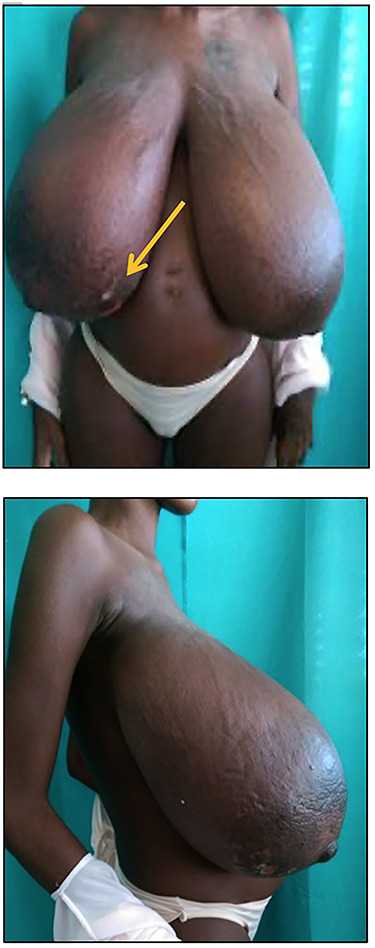
Photograph showing progressive enlargement of bilateral gigantomastia. Note the distended superficial veins and local ulceration to the lower inner quadrant of the right breast (arrow).

The second trimester was complicated by cellulitis to both breasts requiring admission to hospital, frequent local wound care, antibiotics, anti-inflammatories and analgesics. She continued to have progressive enlargement of both breast (left being bigger than the right) with worsening of the initial symptoms. Examination revealed a relatively slim woman with massively enlarged breast bilaterally with local signs of cellulitis. She had grade 3 ptosis, and widening of the nipple areolar complex. The sternal notch to nipple distance measured 45 and 48 cm on the right and left, respectively (Fig. 2).

In the third trimester, her breasts continued to increase in size with small areas of ulcerations noted. The patient became significantly overwhelmed emotionally and reported that she was unable to cope as the symptoms were limiting her daily activities. Following a multidisciplinary team meeting with the obstetricians, neonatologists, anaesthesiologists, plastic surgeons and psychiatrist, a decision was made to improve foetal lung maturity with parenteral steroids and deliver her at a GA of 33/40 via lower segment caesarean section. A live male infant was delivered with appearance, pulse, grimmace, activity and respiration (APGAR) score of 8 (at delivery) and 9 (5 min post-delivery) and a birth weight of 1.55 kg.

**Figure 3:**
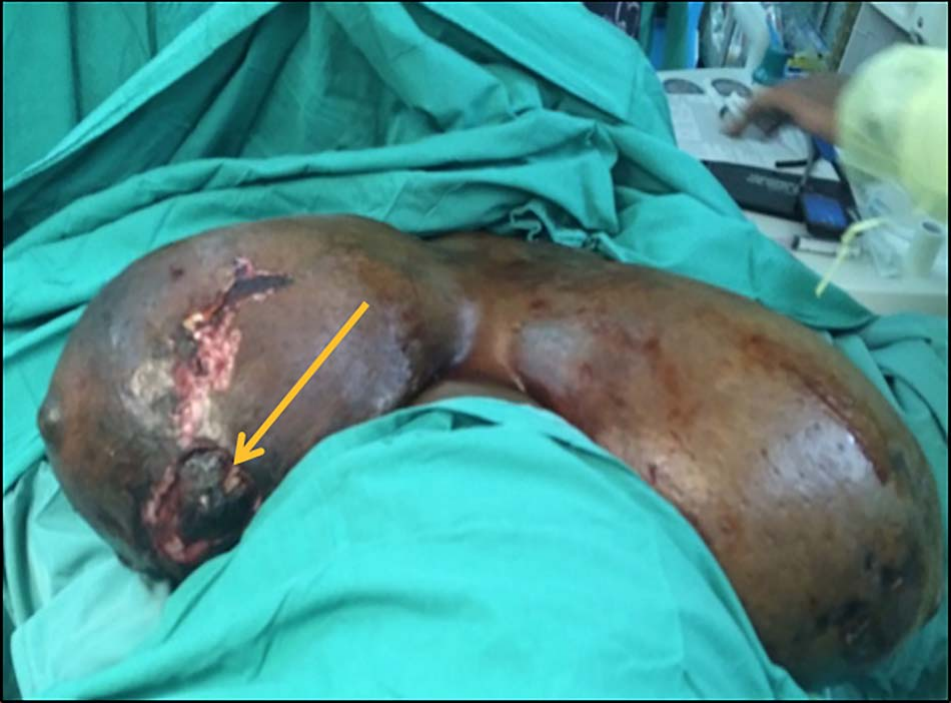
Intraoperative findings showing significant ulceration to lower inner aspect of right breast. Note the clot to the larger ulcerated area (arrow).

However, on postpartum day 4, spontaneous haemorrhage was noted from an ulcer on the right breast (Fig. 3), resulting in significant blood loss (Grade 3 shock) warranting blood transfusion. Her haemoglobin dropped from 7.2 to 4 g/dl. The bleeding was temporized using suture ligation and compression. The patient was taken to the operating theatre where a right unilateral total mastectomy was performed. The specimen weighed 2940 gm. Two weeks later, following a thorough discussion with the patient, an elective contralateral total mastectomy was undertaken with specimen weighing 4750 gm.

**Figure 4:**
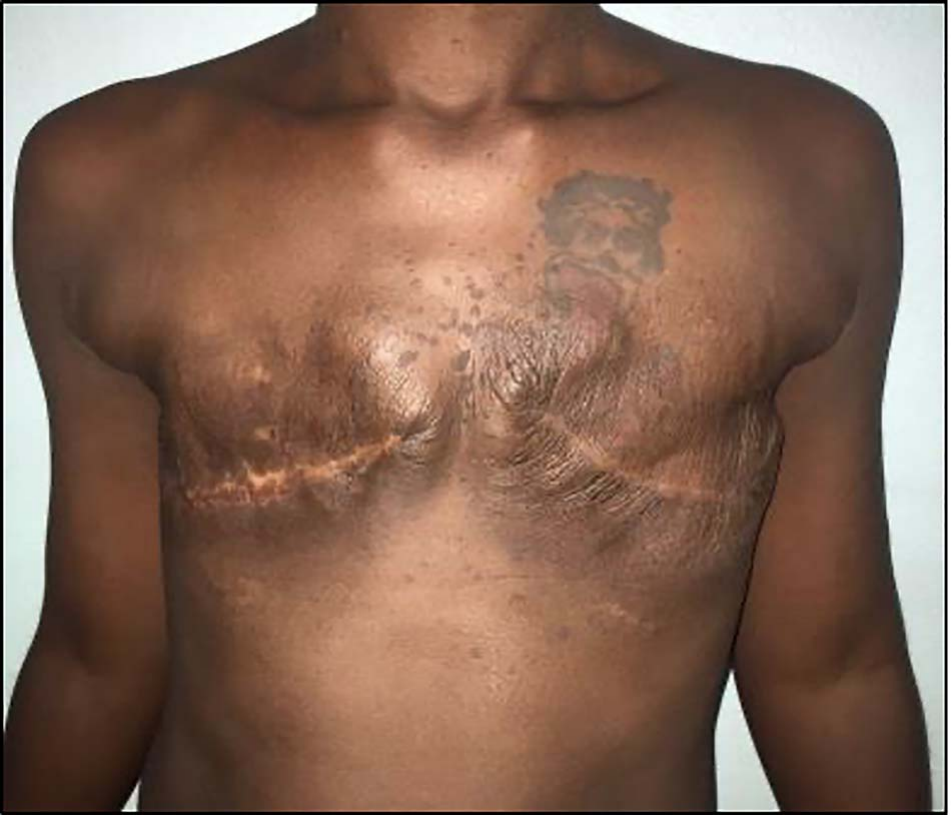
Photograph showing patient 2 months post-operation following bilateral simple mastectomy.

**Figure 5:**
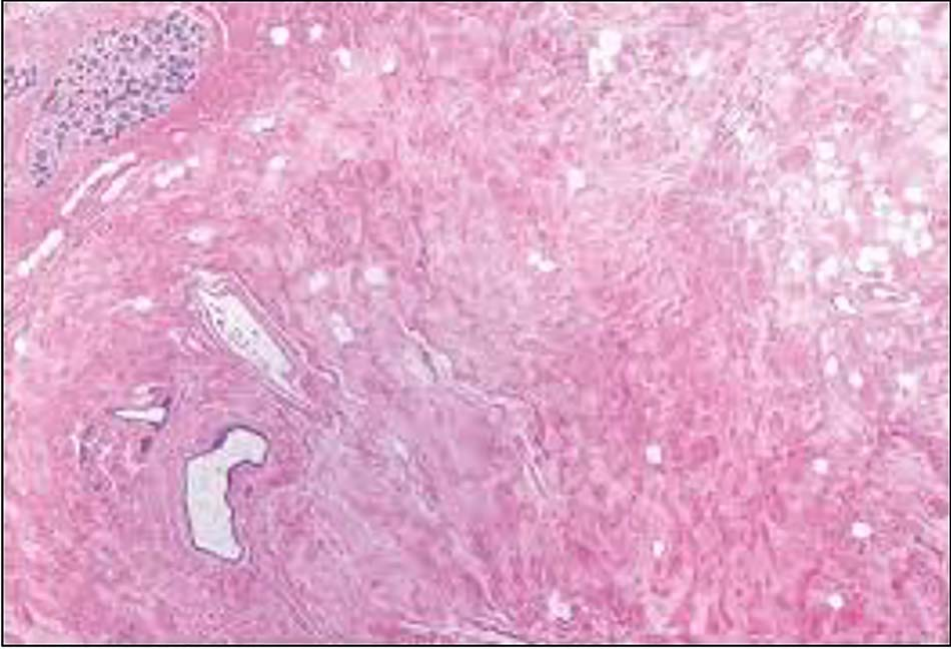
Histological slide showing moderately oedematous interlobular stroma surrounding a normal lobule with lymphangiectasia.

The patient had an uncomplicated recovery period. Histopathological examination of the specimens concluded bilateral GG with lymphangiectasia (Fig. 5). She was counselled on, but declined delayed breast reconstruction, as she was symptom free and otherwise satisfied with the outcome (about 2 months post-surgery) (Fig. 4). Her infant had no significant milestone delays after a short admission period in the special care nursery.

## DISCUSSION

GG is a rare clinical condition resulting in significant breast growth during pregnancy [4]. The aetiology, risk factors and pathogenesis of GG remains difficult to elucidate. Postulated theories include an increase in placental hormones triggering an increase in breast growth as noted in the index case [1]. Other hypotheses include increase breast receptor sensitivity and underlying autoimmune disease [2, 3, 5, 6]. Incidence is noted to be highest in first trimester, in Caucasians and multiparous women with prior history of GG [2].

GG may lead to significant debilitating symptoms including back and shoulder pain, shortness of breath and local fungal infection (intertrigo). In addition to the physical effects, psychological trauma (including depression and social phobia) may occur and can directly impact the pregnancy. The index case had all these physical and psychological complications and the massive breasts caused difficulties with ambulation, leading to the need of a wheelchair. Life-threatening haemorrhage in GG is rarely reported. In a review of the literature, only one similar case of massive haemorrhage was noted, with the patient having an unfavourable outcome despite several transfusions and attempts breast tissue retention [7].

The treatments for GG are not standardized and are implemented on a case-by-case basis with two approaches of medical and/or surgical management. Medical management is implemented with an aim to improve the adverse conditions affecting the pregnancy by addressing both the physical and psychological needs of the patient. This modality is successfully in 39% of cases reported in the literature [8]. Other options such as termination of the pregnancy was chosen by 8.7% of mothers with GG and 10% had spontaneous abortions [8].

However, surgery in the form of a reduction mammoplasty or a total mastectomy is warranted when medical management fails, patient develops significant debilitating or life-threatening disease such as the index case [8]. Reduction mammoplasty confers better aesthetic outcomes and the potential ability to breast feed, but may result in an increased risk of recurrence [9]. In the index case, a total mastectomy was performed as a lifesaving procedure to mitigate further blood loss, which also eliminates the risk of future recurrence. Surgeons should be aware of this life-threatening complication in this relatively rare condition.
